# Modulation of PI3K signaling to improve CAR T cell function

**DOI:** 10.18632/oncotarget.26334

**Published:** 2018-11-09

**Authors:** Wenting Zheng, Lindsay L. Jones, Terrence L. Geiger

**Affiliations:** Department of Pathology, St. Jude Children's Research Hospital, Memphis, TN, USA

**Keywords:** immunotherapy, CAR-T cell, PI3K, T cell receptor, cancer

During CD8^+^ T cell activation, engagement of the T cell receptor (TCR) along with costimulatory receptors triggers signaling pathways that lead to T cell expansion and differentiation. Among these, activation of phosphoinositide 3-kinase (PI3K) has a critical effect on T cell proliferation, survival, migration, and effector/memory subset formation. Class I PI3Ks are composed of one of three isoforms of the p110 catalytic subunit (p110α, p110β, or p110δ), that constitutively associate with a p85 regulatory subunit. Class I PI3Ks catalyze the phosphorylation of phosphatidylinositol 4,5-bisphosphate, generating phosphatidylinositol (3,4,5)-trisphosphate, which recruits proteins containing pleckstrin homology (PH) domains to the plasma membrane. PH domain-containing targets, including AKT, initiate signaling and activate downstream effectors of cellular differentiation and metabolism [1]. During T cell activation, signaling through the T cell receptor, costimulatory molecules, and IL-2 receptor can all activate PI3Kδ. TCR ligation induces zeta-chain associated ZAP70-mediated phosphorylation of LAT, which is required for the recruitment of PI3K to the membrane [2]. The costimulatory molecules CD28 and ICOS contain the consensus YxxM PI3K binding motif in their cytoplasmic tails. The mechanism of PI3Kδ activation through IL-2 signaling may involve LCK/FYN activity and control of the accumulation of phosphatidylinositol (3,4,5)-trisphosphate [3]. PI3Kδ signaling after T cell activation leads to AKT-dependent inactivation and nuclear exclusion of FOXO1, which has been implicated in the downregulation of memory T cell markers such as IL-7Rα and CD62L. PI3Kδ also promotes mTOR signaling, leading to increased T cell metabolic activity which facilitates effector T cell differentiation and function [2].

While loss of PI3K activity is detrimental to immune function, constitutive activation of PI3K also impairs immunity because it preferentially promotes formation of short-lived terminally differentiated effector T cells at the expense of long lived memory T cells. Control of T cell activation and differentiation by PI3K is particularly relevant to Chimeric Antigen Receptor (CAR) T cell immunotherapy. CARs retarget genetically modified T lymphocytes through hybrid receptors that incorporate a tumor antigen-specific scFv, one or more costimulatory domains (most commonly 41BB or CD28), and the CD3-zeta domain. CAR-T cells have experienced a surge in interest due to the now proven effectiveness of CD19-specific CAR-T cells in the treatment of precursor B cell malignancies. CAR-modified T cells are not merely retargeted conventional T lymphocytes. The presence of a CAR on a T cell's surface alters its activation and differentiation, even in the absence of a complementary ligand. Constitutive self-signaling through CAR, related to both the scFv framework and the signaling domains, can lead to aberrant T cell behavior, including altered differentiation and decreased survival. This is significant as the effectiveness of CAR-T cells in patients is directly associated with their *in vivo* longevity. Long *et. al.* demonstrated that the presence of the CD28 costimulatory domain increased CAR-T cell exhaustion induced by persistent CAR self-signaling; the 4-1BB costimulatory domain had a lesser effect [4]. Using a panel of mutant CAR, our group identified a dominant role of the CAR CD3-zeta ITAMs in self-signaling. CD3-zeta significantly enhanced the constitutive activation of the PI3K, AKT, mTOR, and glycolysis pathways, and fostered formation of short-lived effector cells over central/stem memory cells [5].

Manipulation of PI3K signaling can be used to prevent altered CAR-T cell differentiation due to constitutive CAR self-signaling and foster long-lived memory T cell development. We demonstrated that pharmacologic blockade of PI3K during CAR-T manufacture and *ex vivo* expansion abrogated preferential effector T cell development and restored the CAR-T effector/memory ratio to that observed in empty vector transduced T cells. This improved in *vivo* T cell persistence and therapeutic activity in an AML model. Inhibition of p110δ PI3K has also been found to enhance efficacy and memory in tumor-specific therapeutic CD8 T cells, while inhibition of p110α PI3K increased cytokine production and antitumor response [6, 7].

**Figure 1:**
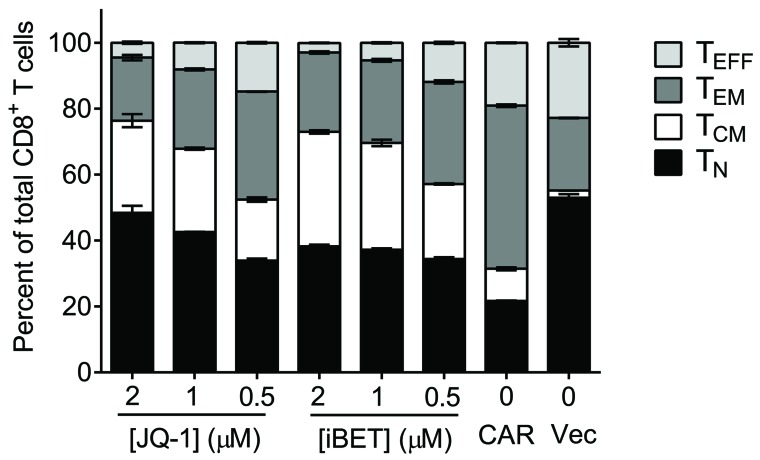
c-Myc inhibition restrains aberrant CAR-T cell differentiation ex vivo T cells were activated with anti-CD3/anti-CD28 and transduced with CD33 CAR or empty vector. Five days after initial activation, CD33 CAR-T cells were treated with c-Myc inhibitors JQ-1 or iBET as indicated for four days. Percentages of T_N_ (naïve; CCR7^+^CD45RA^+^), T_CM_ (central memory; CCR7^+^CD45RA^–^), T_EM_ (effector memory; CCR7^–^CD45RA^–^) and T_EFF_ (effector; CCR7^–^CD45RA^+^) subsets of CD8^+^ T cells were determined by flow cytometry. Results indicate that the bromodomain inhibitors increase the proportion of longer-lived naïve and central memory phenotype T cells.

Downstream targets of PI3K include AKT, mTOR, and FOXO1, and are important in determining CD8^+^ T cell fate. Sustained AKT activation leads to T cell terminal differentiation. Its inhibition in CAR modified T cells results in an early memory phenotype and improved antitumor efficacy [8]. In our study, pharmacologic inhibition of AKT, mTOR, or glycolysis during *ex vivo* expansion of CAR-T cells promoted memory over effector cell formation. However, inhibition of these pathways also reduced CAR-T cell proliferative capacity, limiting therapeutic cell expansion [5]. These targets may therefore be suboptimal for inhibiting terminal effector differentiation, and will require further evaluation. PI3K/AKT signaling can also promote c-myc activity by inhibiting c-myc phosphorylation by GSK-3β and its subsequent proteosomal degradation. Inhibition of c-myc by BET bromodomain inhibitors resulted in expansion of CD62L^+^CCR7^+^ T cells with T_N_ and T_CM_ phenotypes, and adoptive transfer of inhibitor-treated CAR T cells extended survival in an ALL model [9]. We have also observed similar preservation of naïve and memory over effector phenotype in AML-specific CAR-T cells after treatment with BET bromodomain inhibitors (Figure 1). Recently, Singh *et. al.* found that the B cell adaptor for PI3-kinase (BCAP) is an important regulator of CD8^+^ effector and memory T cell differentiation, highlighting yet another potential target in the PI3K pathway to balance effector and long-lived memory T cell generation [10]. It remains to be determined whether this or other downstream targets of PI3K will be effective in supporting therapeutic T cell survival and potency. Clearly, multiple studies now indicate that modulation of PI3K and its downstream targets is a promising approach to improve CAR-T cell efficacy by limiting CAR self-signaling effects and improving T cell memory formation, survival, and function. Optimizing the use of inhibitors of these pathways for clinical application is the next challenge.
